# Pulmonary vasodilator use in very preterm infants in United States children’s hospitals

**DOI:** 10.21203/rs.3.rs-5492163/v1

**Published:** 2024-12-05

**Authors:** Nicolas Bamat, Tomas Vega, Matthew Huber, Erik Jensen, Catherine Avitabile, Scott Lorch, Kathleen Gibbs, Michael O’Byrne, David Frank, Nicolas Bamat

**Affiliations:** The Children’s Hospital of Philadelphia; The Children’s Hospital of Philadelphia; The Children’s Hospital of Philadelphia and the University of Pennsylvania; Children’s Hospital of Philadelphia; Children’s Hospital of Philadelphia; The Children’s Hospital of Philadelphia; Children’s Hospital of Philadelphia; The Children’s Hospital of Philadelphia

**Keywords:** infant, premature, preterm, bronchopulmonary dysplasia, chronic lung disease, pulmonary hypertension, inhaled nitric oxide, sildenafil, bosentan, vasodilator

## Abstract

**Objectives:**

To describe common pulmonary vasodilators (PV), exposure timing, and characteristics associated to their use in very preterm (VP) infants.

**Study Design:**

Observational study of VP infants discharged from U.S. children’s hospitals (2011–2021). PV exposures during hospitalization were identified, and multivariable modeling determined characteristics associated with exposure.

**Results:**

Among 37,428 infants, 6.3% received PV. Early inhaled nitric oxide (iNO) and late sildenafil were most common. Early exposure was associated with lower gestational age, aOR: 9.2 (7.3–11.7), 22–25 vs. 29–31 weeks) and small for gestational age (SGA), 2.3 (2.0–2.7). Late exposure was associated with bronchopulmonary dysplasia (BPD) grade, 26.2 (16.8–40.9), grade 3 vs. no BPD) and early PV exposure, 3.7 (2.9–4.8).

**Conclusions:**

Early iNO and late sildenafil are used in VP infants despite limited evidence. Prospective early studies enrolling extremely preterm or SGA infants and late studies enrolling infants with early PV exposure or high-grade BPD would target current evidence gaps.

## Introduction

Complications of preterm birth remain a leading cause of death among children younger than 5 years of age worldwide(1). Most complications have declined over the past decades, except for those relating to lung disease, which remain the primary cause of death among neonates(2, 3). Lung disease-related pulmonary hypertension (PH), including bronchopulmonary dysplasia-associated PH (BPD-PH), is the fastest growing group of pediatric PH(2, 3). Arrested lung development, abnormal postnatal vascular remodeling, and vascular growth arrest can contribute to increased pulmonary vascular resistance and pulmonary arterial hypertension(4). Without treatment, PH can result in right ventricular (RV) heart failure and is associated with a four-fold increase in mortality(4–12). However, there are few FDA-approved treatments for pediatric PH and limited clinical trial data for treating BPD-PH.

Observational studies support pulmonary vasodilator (PV) treatment for infants with persistent lung disease related-PH and RV dysfunction despite optimal management of lung disease, yet the safety and efficacy of pharmacotherapies are incompletely studied(8, 13–16). Despite their common use, prospective studies confirming their safety, efficacy and optimal use are still needed. For example, there is an ongoing National Institutes of Health (NIH) trial comparing the efficacy of sildenafil with that of sildenafil and bosentan combined, which is among the first to include both pulmonary arterial hypertension and lung-disease-related pulmonary hypertension(17). Characterizing PV administration in very preterm infants at risk of, or with BPD-PH is essential to identify the research questions most relevant to current practice (18). Describing temporal characteristics of PV exposures can inform windows for enrollment and treatment in prospective studies and trials. Additionally, identifying characteristics that place infants at high risk of receiving PV can help identify target study populations.

Given the need to further study effective PV strategies in preterm infants, we sought to describe contemporary practice patterns of PV use in a large cohort of very preterm infants admitted to United States children’s hospitals, with a focus on identifying the most common PV exposures, temporal characteristics of their use, and clinical characteristics associated with exposure.

## Methods

### Study Design and Data Source

We conducted a retrospective cohort study using The Children’s Hospital Association Pediatric Health Information System (PHIS) database (Shawnee Mission, KS). The PHIS database contains deidentified administrative and billing data, including patient demographic characteristics, medication administration by hospitalization day, diagnosis and procedure codes, from pediatric hospitals in the US. PHIS data have been reviewed for quality and reliability by participating hospitals and the Child Health Corporation of America and have been used for various analyses of pediatric healthcare utilization in recent years(11, 19–24). The Institutional Review Board of the Children’s Hospital of Philadelphia has a standing policy that studies using PHIS are exempt from review under the Common Rule.

### Study Population

The cohort identification owchart is depicted in Supplementary Figure S1. We included infants born between 22- and 31-weeks gestational age (GA) that were discharged from a PHIS neonatal intensive care unit (NICU) between January 1, 2011 and December 31, 2021. We excluded all infants from hospitals with more than 50% missing data for GA or inconsistent GA data. We excluded infants with a length of stay less than 7 days and those discharged prior to 28 weeks PMA without any documented respiratory support at discharge, as we deemed this implausible. As we considered BPD to be an important associated condition for PH in our population, we excluded infants for whom a BPD classification could not be ascertained. This included infants that were discharged or transferred on respiratory support prior to 36 weeks PMA, died prior to 36 weeks PMA or were admitted after 36 weeks PMA. We excluded subjects with risk factors for developing PH from distinct underlying disease mechanisms for the following reasons: congenital diaphragmatic hernia and/or lung aplasia or hypoplasia, chromosomal abnormalities, and congenital heart disease other than patent ductus arteriosus (PDA), atrial septal defects (ASD), and ventricular septal defects (VSD). Infants were excluded based on the presence of ICD diagnostic codes consistent with the above-mentioned conditions (Supplementary Table S1).

### Study Measures and Variables

Medications classified as PV for our study population were determined by two pediatric cardiologists and co-authors (C.M.A, D.B.F.) and were identified by their generic names in PHIS. These were epoprostenol, iloprost, treprostinil, sildenafil, tadalafil, bosentan, macitentan, ambrisentan, selexipag, riociguat, and inhaled nitric oxide (iNO). We calculated two measures of PV exposure: any study period exposure and cumulative exposure. We defined any study-period exposure as prescription of a PV at any time during the study period, defined for each infant as the time between admission and the first of either discharge or one year of age. We defined cumulative exposure as the total number of study period days in which a PV was prescribed, irrespective of the number of distinct PV prescribed on that day, divided by the total number of subject-days during the study period, reported as a percentage of subject-days. Both measures of PV exposure were calculated for any PV (all the above medications considered jointly), each specific PV, and PV combination therapies, for the duration of the study period. Combination therapy was defined as the concomitant receipt of two PV on the same calendar day. Temporal characteristics of PV exposures were summarized for all PV medications, each specific PV medication, and for the three most common combination therapies as the post-menstrual and chronologic ages at first and last exposure and longest contiguous course duration.

Due to the bimodal distribution of PV exposures, we modeled characteristics associated with any study-period PV exposure and cumulative exposure before 28 days chronologic age (“early”) and those associated with exposure after 36 weeks PMA (“late”) separately. Baseline infant characteristics present at birth were considered in the early PV exposure model, and all characteristics, including early PV exposure, were considered for the late PV exposure model. Variables included gestational age in completed weeks at birth, birth weight, sex, ethnicity, race, BPD grade, small for gestational age (SGA) status, the presence of ventricular septal defects, a patent ductus arteriosus undergoing ligation or occlusion, grade 3 or 4 interventricular hemorrhage, and stage 2 or 3 necrotizing enterocolitis. Race and ethnicity classifications reflect designations made in local hospital data repositories prior to data transfer to PHIS. Ascertainment methods are uncertain and likely vary by hospital. We considered these variables plausibly associated with BPD and/or PH based on past literature, recognizing that associations between race, ethnicity, and these conditions likely represent disparities in healthcare and social determinants of health rather than intrinsic physiologic differences. We chose not to evaluate ASDs as a characteristic in our study due to difficulty distinguishing ASD from physiologic patent foramen ovale based on ICD codes(25). BPD classifications were defined per the 2019 NRN definition based on the highest level of respiratory support used at 36 weeks PMA, identified through the presence of daily clinical service codes(26). We identified infants as SGA if their birth weights were lower than the sex-specific 10th percentile for their gestational age at birth, using values derived from the 2013 revised Fenton Preterm Growth Chart(27). The remaining variables were determined through the presence of qualifying procedure or diagnostic ICD codes (Supplementary Table S2).

### Statistical Analyses

We described study variables with summary statistics, including counts (percentages) and medians [interquartile range (IQR)]. To evaluate the association between clinical characteristics and any study-period PV exposure, we used logistic regression with center as a fixed effect and applying cluster-robust variance estimates to account for the nesting of infants within hospitals, in both unadjusted bivariable and adjusted multivariable models. Characteristics associated with PV exposure in bivariable analyses at *p* < 0.20 were included as covariates in a multivariable model testing the adjusted association between clinical characteristics and PV exposure. Birth weight was described for the cohort but excluded a priori from analyses in favor of gestational age group and SGA status to avoid collinearity. After noting a bimodal distribution in the timing of PV exposures, we chose to model early (< 28 days chronologic age) and late (> 36 weeks PMA) exposures separately. We used an analogous statistical approach for PV cumulative exposure among exposed infants but used a multivariable generalized linear model with logit link and a binomial distribution to model proportions(28). To facilitate clinical interpretation, estimates of the association between predictor variables and outcomes were reported as post-estimation marginal means. We considered *p* < 0.05 to be statistically significant throughout without adjustment for multiple comparisons. All analyses were performed with Stata 16 (StataCorp, College Station, Texas, USA).

## Results

We identified 37 428 eligible infants. Cohort characteristics are detailed in Table 1. Median length of stay during the study period was 67 days [IQR 44–100]. We identified 2 358 (6.3%) infants exposed to a PV during the study period (Table 2). Inhaled nitric oxide (n = 2 160, 5.8%) was the most common exposure, followed by sildenafil (n = 599, 1.6%) and bosentan (n = 60, 0.2%). Exposures to epoprostenol, tadalafil, treprostinil, and iloprost were infrequent (0.1% of cohort or less), while no exposures to macitentan, selexipag, or riociguat were identified. Sildenafil with iNO was the most common combination (n = 340, 0.91%), followed by bosentan with sildenafil (n = 52, 0.14%) and bosentan with iNO (n = 46, 0.12%).

Among infants exposed to a PV, exposures were present for a median of 7.7% of study period days, though this value was right-skewed, with over a quarter of infants exposed for greater than 25% of study days; IQR [3.1–25.3%]. Despite iNO being the most common PV exposure, treatment occurred earlier and for a shorter duration. The median age of first exposure to iNO was 28.4 weeks PMA and 2 weeks chronologic age, and a quarter of iNO exposures were initiated at or prior to 26 weeks PMA and 2 weeks chronologic age.

For all other assessed PV, median ages of first exposure were > 40 weeks PMA and > 16 weeks chronologic age. This resulted in a bimodal distribution for exposure timing, with an early peak before 28 days chronologic age driven by iNO and a later peak after 36 wk PMA driven by sildenafil. Supplementary Figure S2 depicts the percentage of infants exposed to any PV, iNO, sildenafil and bosentan as a function of PMA. There were notable differences in course duration between specific medications as well. Infants were exposed to iNO for less than 6% of study period days, while median exposures to sildenafil, tadalafil, and bosentan were all for greater than 20% of study period days.

Clinical characteristics associated with early PV exposure are shown in Table 3. The characteristics most strongly associated with early PV exposure were lower GA and SGA status. Infants born at a lower GA were more likely to have early PV exposure compared to infants born at 29–31 wk GA, with the highest odds noted in the most immature (22–25 weeks) GA group; adjusted odds ratio (aOR) (95% CI): 9.2 (7.3, 11.7), p < 0.001. Infants born SGA were more likely to have early exposure compared to non-SGA infants; aOR (95% CI): 2.3 (2.0, 2.7.), p < 0.001. Weaker, yet statistically significant associations were: early PV exposures in males compared to females and lower odds in Asian compared to White infants.

Clinical characteristics associated with late PV exposure beyond 36 wk PMA are shown in Table 4. The strongest association was with BPD grade, for which we identified a statistically significant (p < 0.001) increased odds relative to no BPD for each grade, and a large stepwise increase in odds with each progressive grade. Infants with grade 3 BPD had an aOR (95% CI) of 26.2 (16.8, 40.9) compared to no BPD. Early PV exposure was the second strongest predictor of late PV exposure; aOR (95% CI): 3.7 (2.9, 4.8), p < 0.001. Both lower gestational age and SGA status were strongly associated (aOR > 2.0, p < 0.001) with greater odds of late PV exposure, and weaker, yet statistically significant associations were noted for the presence of a VSD and a PDA undergoing procedural closure, both associated with higher odds. Among the 2,358 infants exposed to a PV, the clinical characteristics most strongly associated with a greater extent of cumulative exposure were grade 3 BPD and SGA status (Table 5).

## Discussion

As survival following very preterm birth increases, rates of lung disease and associated PH will follow. Pulmonary vasodilators are commonly used promising pharmacotherapies in these infants, yet clinical trial data to confirm their safe and effective use are limited(13, 14, 16, 17, 29–32). We sought to characterize contemporary PV use, temporal characteristics of their use, and identify characteristics of very preterm infants at highest risk of exposure to inform future research.

We identified iNO and sildenafil as the most common PV exposures, with notably lower use for all other PV. The proportion of cohort infants exposed to iNO and sildenafil was 29-fold and 8-fold higher, respectively, than for bosentan, the next most common PV exposure. The percentage of cohort infants exposed to iNO (5.8%) and sildenafil (1.6%) in our cohort were broadly consistent with previous observational studies of similar populations(22, 24). The use of other PV medications, either individually or in combination, is currently relatively rare. A small number of trials have studied iNO or sildenafil for prevention or treatment of BPD-PH(33–35). An ongoing trial is evaluating initiating combined sildenafil and bosentan versus sildenafil alone(17). Our findings highlight the importance of further studying sildenafil in preterm infants with BPD-PH and opportunities to conduct research to inform use prior to loss of equipoise for other PV medications. Our findings also highlight the continued common early use of iNO in preterm infants despite a lack of supportive evidence from clinical trials(36), highlighting the potential for future studies to address this discordance.

We identified distinct temporal use patterns between medications, with iNO predominating early in the postnatal course and continuing to some degree at later stages, when sildenafil becomes more prominent. Median ages of initiation and course durations for both iNO and sildenafil were also broadly consistent with prior observational studies in similar populations(22, 24, 30, 37). While iNO use is approved by the U.S. Food and Drug Administration for the management of persistent pulmonary hypertension of the newborn (PPHN) in term and near-term infants, iNO prevention and/or treatment for PH related to premature lung disorders lacks conclusive evidence(36, 38). A 2017 Cochrane review reported effect estimates that approached significance for the outcome of less death or BPD with iNO over placebo in preterm infants and recommended further study(36), however the subsequent NEWNO trial found no benefit on survival without BPD or neurodevelopmental outcomes(35). The median PMA for iNO use in our cohort was 28 weeks, with a quarter being exposed at or before 26 weeks, highlighting ongoing use of iNO use in very immature preterm populations. The gradual increase and later peak in PH-targeted therapies was primarily driven by sildenafil. Sildenafil and bosentan were both initiated at a median PMA beyond 40 weeks PMA and 4 months chronologic age and were administered for prolonged durations. A possible explanation for these temporal exposure patterns may be the natural progression of cardiopulmonary pathology, including BPD-PH, and increased echocardiographic screening for this condition among infants with BPD at 36 weeks PMA, per guideline recommendations (13, 14, 16, 37). Few prospective trials have assessed the pharmacology and efficacy of sildenafil in preterm infants with established BPD, with the current best evidence derived from retrospective cohorts (39). A 2022 phase I open-label trial sought to establish the safety of sildenafil in preterm infants ≤ 32 weeks GA(40). The same group is currently enrolling for the SILDI-SAFE phase II trial, which will provide safety, pharmacokinetics, and preliminary effectiveness data for BPD prevention in younger at-risk preterm infants. Our data highlight the need for rigorous study of sildenafil in older preterm infants with established BPD such as in the ongoing Kids ModPAH trial (NCT #04039464).

The characteristics associated with early exposure included well-established risk factors for PH, including greater prematurity and SGA status, both associated with incomplete and arrested lung development, abnormal postnatal vascular remodeling, and vascular growth arrest(4). A lower GA and SGA status were also significantly associated with late PV exposure. We noted a large stepwise increase in late PV exposure with progressive BPD grade, particularly in the presence of grade 3 BPD. Further, grade 3 BPD was strongly associated with the extent of cumulative PV use among exposed infants. To our knowledge, this is the first study reporting an association between BPD grade and PV exposure using the 2019 NRN Jensen classification system, emphasizing infants with ongoing mechanical ventilation at 36 weeks PMA as a high-risk population for PH and a target population for study(26). We also identified late PV exposure to be strongly associated with early PV exposure, supporting previous reports that early echocardiographic evidence of pulmonary vascular disease may be associated with the development of BPD and late PH.(41, 42)

We found weak but statistically significant associations between race and PV exposure, the strongest of which was odds of being exposed to PV beyond 36 weeks PMA among black compared to white infants. These associations likely reflect multifactorial social determinants contributing to disparities in neonatal respiratory outcomes(43).

We demonstrated associations between a present VSD and PDA undergoing surgical or catheter-based intervention with late PV exposure. Increased pulmonary blood flow from left-to-right intracardiac shunt can cause endothelial damage, adverse pulmonary vascular remodeling, and increased pulmonary vascular resistance. This process usually takes several months to develop but may be more injurious in preterm patients with a limited pulmonary vascular bed^44^. However, it is uncertain if this association reflects causal physiologic mechanisms or residual confounding bias as sicker infants are more likely to undergo PDA closure and considered for PH screening and treatment(44).

Our study has important limitations. We were unable to determine the precise indications for PV use and the timing of their use relative to the establishment of diagnoses, which limits our ability to more fully describe PV prescription. Our data are derived from an administrative database in which many study characteristics (e.g. PDA with procedural closure, VSD, etc.) were derived from ICD codes, which are based on the accuracy of provider documentation, making them susceptible to misclassification bias. Further, we could only ascertain the dichotomous presence or absence of some characteristics, without a measure of severity. We lacked granular information regarding medication exposure characteristics such as dose and route of administration. We lacked information on exposures that occurred in the window between birth and admission to PHIS hospitals, and we also excluded infants who died before a BPD classification was possible. Both of these limitations likely contribute to an underreporting of some medication exposures, such as early iNO use. Lastly, as PHIS reports data from children’s hospitals, findings from our cohort may not generalize to broader very preterm populations. Carefully planned prospective studies would be able to overcome many of the current limitations..

We provide detailed information on the most common PV medications, timing of their use, and infant characteristics associated with exposure. Inhaled iNO and sildenafil were the most common PV exposures despite a lack of supportive clinical research evidence, emphasizing the importance of future trials assessing their safety and efficacy. Our study identifies infants born extremely preterm and with growth restriction as key target populations for early PV trials, and infants with early PV exposure and evolving or established high grade BPD as the most relevant populations for late PV trials as preterm infants approach term-corrected age. Our study suggests that high quality research in these population would best address the ongoing gap between contemporary clinical practice and research evidence.

## Figures and Tables

**Table 1:** Cohort characteristics

Characteristic	*N* = 37428
Gestational age, completed weeks, n(%)	
22–25	7303 (20)
26–28	12000 (32)
29–31	18125 (48)
Sex, n(%)	
Female	17398 (46)
Male	20022 (54)
Ethnicity, n(%)	
Not Hispanic or Latino	27945 (75)
Hispanic or Latino	6366 (17)
Other or unknown	3117 (8)
Race, n(%)	
White	19068 (51)
Black	10356 (28)
Asian	1319 (4)
Other or unknown	6685 (18)
Small for gestational age, n(%)	2793 (8)
Ventricular septal defect, n(%)	2262 (3)
PDA with procedural closure, n(%)	2816 (8)
BPD grade^1^, n(%)	
No BPD	27108 (72)
Grade 1	5167 (14)
Grade 2	2565 (7)
Grade 3	2588 (7)
Grade 3 or 4 IVH, n(%)	3378 (9)
Stage 2 or 3 NEC, n(%)	3708 (10)

Abbreviations: PDA, patent ductus arteriosus, BPD, bronchopulmonary dysplasia, IVH, intraventricular hemorrhage, NEC, necrotizing enterocolitis

1 Based on Jensen/Neonatal Research Network 2019 classification

**Table 2:** Pulmonary vasodilator exposures and temporal characteristics of their use in very preterm infants

Pulmonary	Any study period	Percentage of days	First exposure,	Last exposure,	First exposure,	Last exposure,	Longest course
Vasodilator^1^	Exposure^2^, n (%)	with PV exposure, median, % (IQR)^3^	PMA weeks, median (IQR)	PMA weeks, median (IQR)	Chronologic weeks, median (IQR)	Chronologic weeks, median (IQR)	duration, days, median (IQR)^4^
Any PV	2,358 (6.3)	7.7 (3.1–25.3)	29.0 (26.3–34.3)	31.4 (28.0–43.6)	2.4 (0.4–8.4)	3.9 (1.0–9.1)	8 (4–27)
Inhaled Nitric Oxide	2,160 (5.8)	5.7 (2.6–12.7)	28.4 (26.1–32.0)	30.6 (27.6–38.0)	2.0 (0.3–6.3)	4.1 (1.1–10.3)	6 (3–14)
Sildenafil	599 (1.6)	37.1 (18.7–54.0)	41.0 (36.4–46.3)	53.4 (44.4–64.7)	16.0 (11.1–21.0)	21.8 (14.4–31.6)	64 (27–117)
Bosentan	60 (0.2)	20.9 (4.3–37.2)	49.9 (43.4–55.5)	57.2 (49.9–72.4)	24.2 (17.9–30.6)	31.9 (21.9–37.3)	36 (8–103)
Epoprostenol	26 (0.1)	4.9 (3.0–7.1)	42.5 (33.6–52.7)	45.0 (35.3–55.7)	17.9 (8.9–27.7)	20.1 (5.2–32.2)	7 (2–15)
Tadalafil	15 (0.04)	45.6 (22.9–56.8)	45.0 (41.9–50.0)	55.7 (50.7–65.3)	20.9 (12.9–23.9)	28.6 (24.4–37.7)	80 (41–149)
Treprostinil	14 (0.04)	20.3 (10.1–30.3)	45.6 (40.9–48.3)	61.8 (47.0–74.7)	20.9 (13.7–23.7)	35.6 (20.0–51.7)	3 (2–5)
Iloprost	13 (0.03)	4.2 (2.5–8.4)	40.1 (31.3–50.7)	40.4 (34.6–51.1)	16.1 (4.3–23.7)	12.4 (7.6–22.9)	6 (4–15)
Sildenafil and iNO	340 (0.91)	6.1 (2.7–12.7)	41.1 (36.0–47.0)	46.4 (39.1–54.2)	16.0 (10.1–22.1)	19.9 (12.4–29.0)	12 (5–30)
Bosentan and Sildenafil	52 (0.14)	20.9 (4.3–35.6)	50.2 (43.6–56.3)	57.2 (49.8–74.2)	25.4 (18.3–31.1)	32.6 (24.7–41.0)	38 (8–101.5)
Bosentan and iNO	46 (0.12)	6.6 (2.7–12.1)	49.9 (43.7–56.3)	54.9 (48.4–61.4)	26.2 (18.7–31.9)	32.6 (25.7–35.9)	15 (6–36)

Abbreviations: PV, pulmonary vasodilator(s), PMA, postmenstrual age, IQR, interquartile range.

All values reflect count (percent) or median (interquartile range)

1 Other PV assessed but not identified in the cohort were macitentan, selexipag and riociguat.

2 Proportion of subjects in study cohort (N = 37428) with a PV exposure at any time during study period, defined as between admission and the first of either discharge or one year of age.

3 Proportion of study period days with any PV exposure, per infant, among infants with at least one study period exposure.

4 Longest contiguous period with daily exposure, per infant, among those with at least one study period exposure

**Table 3:** Characteristics associated with early pulmonary vasodilator exposure in very preterm infants

Characteristic	Any early pulmonary vasodilator exposure		
	N (%) 1445/35968 (4.0)	Unadjusted OR (95% CI)	Adjusted OR (95% CI)
Gestational age, completed weeks			
22–25	743/6588 (11.3)	9.0 (7.1, 11.5)^†^	9.2 (7.3, 11.7) ^†^
26–28	458/11463 (4.0)	2.9 (2.5, 3.5)^†^	3.0 (2.5, 3.5) ^†^
29–31 (ref.)	244/17917 (1.4)	-	-
Sex			
Female (ref.)	611/16760 (3.6)	-	-
Male	833/19201 (4.3)	1.2 (1.0, 4.4)*	1.2 (1.1, 1.4)*
Ethnicity			
Hispanic or Latino (ref.)	220/6065 (3.6)	-	-
Not Hispanic or Latino	1123/26901 (4.2)	1.1 (0.9, 1.3)	-
Other or Unknown	102/3002 (3.4)	1.1 (0.8, 1.3)	-
Race			
White (ref.)	731/18419 (4.0)	-	-
Black	473/9929 (4.8)	1.1 (1.0, 1.3)	0.9 (0.8, 1.1)
Asian	35/1269 (2.8)	0.7 (0.5, 0.9)*	0.6 (0.5, 0.9)*
Other or unknown	242/6351 (3.8)	0.9 (0.8, 1.1)	0.8 (0.8, 1.0)
Small for gestational age			
No (ref.)	1194/32466 (3.7)	-	-
Yes	215/2675 (8.0)	2.3 (2.0, 2.6)^†^	2.3 (2.0, 2.7) ^†^

Abbreviations: OR, odds ratio, CI, confidence interval, ref., reference value.

* p-value < 0.05,

† p-value < 0.001

Baseline characteristics present at birth were considered for an association with early (< 28 days chronologic age) PV exposure. Characteristics associated with early PV exposure in bivariable analyses at p < 0.20 were included in multivariable logistic regression models adjusting for center as a fixed effect and using cluster-robust variance estimates to control for nesting of subjects within centers. All characteristics met criteria for multivariable model inclusion except for ethnicity.

**Table 4:** Cohort characteristics associated with late pulmonary vasodilator exposure in very preterm infants

Characteristic	Any late pulmonary vasodilator exposure		
	N (%) 883/37428 (2.4)	Unadjusted OR (95% CI)	Adjusted OR (95% CI)
Gestational age, completed weeks			
22–25	518/7303 (7.1)	15.0 (10.7, 21.2)^†^	3.0 (2.2, 4.2)^†^
26–28	283/12000 (2.4)	5.13 (3.7, 7.0)^†^	2.2 (1.6, 3.0)^†^
29–31 (ref.)	82/18125 (0.5)	-	-
Sex			
Female	377/17398 (2.2)	-	-
Male	506/20022 (2.5)	1.1 (1.0, 1.3)	1.1 (0.9, 1.3)
Maternal ethnicity			
Not Hispanic or Latino	687/27945 (2.5)	1.3 (1.0, 1.7)*	1.2 (0.9, 1.5)
Hispanic or Latino (ref.)	114/6366 (1.8)	-	-
Other or unknown	82/3117 (2.6)	2.0 (1.3, 2.8)^†^	1.8 (1.2, 2.6)*
Race			
White (ref.)	351/19068 (1.8)	-	-
Black	341/2356 (14.5)	1.7 (1.4, 2.0)^†^	1.5 (1.2, 1.8)^†^
Asian	30/1319 (2.3)	1.5 (0.9, 2.3)	1.8 (1.0, 3.5)
Other or unknown	161/6685 (2.4)	1.2 (1.0, 1.6)	1.3 (1.0, 1.6)*
Small for gestational age			
No (ref.)	605/33431 (1.8)	-	-
Yes	208/2793 (7.4)	4.3 (3.7, 5.0)^†^	2.7 (2.2, 3.3)^†^
Ventricular septal defect			
No (ref.)	828/36277 (2.3)	-	-
Yes	55/1151 (4.8)	2.0 (1.5, 2.6)^†^	1.6 (1.1, 2.3)*
PDA with procedural closure			
No (ref.)	611/34612 (1.8)	-	-
Yes	272/2816 (9.7)	5.4 (4.2, 7.1)^†^	1.9 (1.4, 2.5)^†^
BPD grade^1^			
No BPD (ref.)	119/27108 (0.4)	-	-
Grade 1	116/5167 (2.2)	4.6 (2.8, 7.5)^†^	2.7 (1.6, 4.8)^†^
Grade 2	154/2565 (6.0)	16.3 (10.1, 26.3)^†^	9.1 (5.7, 14.5)^†^
Grade 3	494/2588 (19.1)	59.7 (38.3, 93.0)^†^	26.2 (16.8, 40.9)^†^
Grade 3 or 4 IVH						
No (ref.)	707/34050 (2.1)	-	-
Yes	176/3378 (5.2)	2.0 (1.6, 2.5)^†^	0.9 (0.7, 1.0)
Stage 2 or 3 NEC						
No (ref.)	654/33720 (1.9)	-	-
Yes	229/3708 (6.2)	2.5 (1.9, 3.3)^†^	1.0 (0.8, 1.3)
Early PV exposure^2^						
No (ref.)	535/34523 (1.5)	-	-
Yes	200/1445 (13.8)	10.6 (8.7, 12.9)^†^	3.7 (2.9, 4.8)^†^

Abbreviations: OR, odds ratio, CI, confidence interval, ref., reference value, PDA, patent ductus arteriosus, BPD, bronchopulmonary dysplasia, IVH, intraventricular hemorrhage, NEC, necrotizing enterocolitis, PV, pulmonary vasodilator

* p-value < 0.05,

† p-value < 0.001

1 Based on Jensen/Neonatal Research Network 2019 classification

2 Early PV exposure defined as < 28 days chronologic age

All cohort characteristics considered for an association with late (> 36 weeks PMA) PV exposure.

Characteristics associated with late PV exposure in bivariable analyses at p < 0.20 were included in multivariable logistic regression models adjusting for center as a xed effect and using cluster-robust variance estimates to control for nesting of subjects within centers. All characteristics met criteria for multivariable model inclusion.

**Table 5:** Cohort characteristics associated with cumulative exposure to pulmonary vasodilators in very preterm infants exposed to pulmonary vasodilators

Characteristic	Percentage of Study Period Days Exposed	
	Unadjusted Marginal Mean, % (95% CI)	Adjusted Marginal Mean, % (95% CI)
Gestational age, completed weeks		
22–25	19 (18, 19)*	18 (17, 19)
26–28	18 (17, 19)*	17 (16, 18)
29–31 (ref.)	15 (13, 17)	16 (14, 19)
Sex		
Male	18 (17, 19)	-
Female (ref.)	18 (17, 19)	-
Ethnicity		
Not Hispanic or Latino	18 (17, 18)	17 (16, 17)
Hispanic or Latino (ref.)	17 (15, 20)	18 (16, 21)
Other or unknown	23 (18, 28)*	22 (17, 26)
Race		
White (ref.)	17 (16, 18)	17 (16, 18)
Black	19 (17, 20)	18 (17, 20)
Asian	23 (16, 29)	24 (18, 30)*
Other or unknown	18 (16, 21)	17 (14, 19)
Small for gestational age		
No (ref.)	16 (16, 17)	16 (16, 17)
Yes	24 (21, 26)^†^	22 (20, 24)^†^
Ventricular septal defect		
No (ref.)	18 (18, 18)	17 (17, 17)
Yes	21 (17, 25)*	20 (16, 24)
PDA with procedural closure		
No (ref.)	17 (17, 18)	17 (17, 18)
Yes	20 (19, 22)*	18 (17, 20)
BPD grade^1^		
No BPD (ref.)	14 (12, 15)	14 (12, 16)
Grade 1	13 (11, 14)	13 (11, 14)
Grade 2	16 (14, 19)*	17 (14, 19)
Grade 3	25 (23, 28)^†^	24 (21, 26)^†^
Grade 3 or 4 IVH		
No (ref.)	19 (18, 19)	18 (17, 18)
Yes	15 (14, 17)*	16 (14, 17)*
Stage 2 or 3 NEC		
No (ref.)	19 (18, 19)	19 (18, 19)
Yes	15 (13, 17)*	14 (13, 16)^†^

Abbreviations:, CI, confidence interval, PDA, patent ductus arteriosus, BPD, bronchopulmonary dysplasia, IVH, intraventricular hemorrhage, NEC, necrotizing enterocolitis

* p-value < 0.05,

† p-value < 0.001

1 Based on Jensen/Neonatal Research Network 2019 definition,

Cohort restricted to those infants with at least one pulmonary vasodilator exposure during study period, defined as between admission and the first of either discharge or one year of age; N = 2358. All cohort characteristics considered for association with cumulative exposure to pulmonary vasodilators, reported as the percentage of study period days with exposure. Characteristics associated with cumulative exposure in bivariable analyses at p < 0.20 were included in multivariable generalized linear model with logit link and binomial distribution adjusting for center as a fixed effect and using cluster-robust variance estimates to control for nesting of subjects within centers. All characteristics except sex met criteria for inclusion. Estimates of the association were reported as post-estimation marginal means to facilitate clinical interpretation.

## Abbreviations

ASD: atrial septal defect
BPD: bronchopulmonary dysplasia
BPD-PH: bronchopulmonary dysplasia-associated pulmonary hypertension
GA: gestational age
iNO: inhaled nitric oxide
PDA: patent ductus arteriosus
PH: pulmonary hypertension
PMA: postmenstrual age
PV: pulmonary vasodilator
SGA: small for gestational age
VSD: ventricular septal defects

## References

[1] Perin J, Mulick A, Yeung D, Villavicencio F, Lopez G, Strong KL, Global, regional, and national causes of under-5 mortality in 2000–19: an updated systematic analysis with implications for the Sustainable Development Goals. Lancet Child Adolesc Health [Internet]. 2022 Feb 1 [cited 2024 Apr 13];6(2):106–15. Available from: http://www.thelancet.com/article/S2352464221003114/fulltext 34800370 10.1016/S2352-4642(21)00311-4 PMC8786667

[2] Stoll BJ, Hansen NI, Bell EF, Walsh MC, Carlo WA, Shankaran S, Trends in Care Practices, Morbidity, and Mortality of Extremely Preterm Neonates, 1993–2012. JAMA [Internet]. 2015 Sep 8 [cited 2022 Jul 19];314(10):1039–51. Available from: https://jamanetwork.com/journals/jama/fullarticle/2434683 26348753 10.1001/jama.2015.10244 PMC4787615

[3] Manuck TA, Rice MM, Bailit JL, Grobman WA, Reddy UM, Wapner RJ, Preterm Neonatal Morbidity and Mortality by Gestational Age: A Contemporary Cohort. Am J Obstet Gynecol [Internet]. 2016 Jul 1 [cited 2024 May 18];215(1):103.e1. Available from: /pmc/articles/PMC4921282/10.1016/j.ajog.2016.01.004 PMC4921282 26772790

[4] Hansmann G, Sallmon H, Roehr CC, Kourembanas S, Austin ED, Koestenberger M. Pulmonary hypertension in bronchopulmonary dysplasia. Pediatr Res. 2021;89(3):446–55. 32521539 10.1038/s41390-020-0993-4 PMC7979539

[5] Khemani E, McElhinney DB, Rhein L, Andradeb O, Lacro R V., Thomas KC, Pulmonary artery hypertension in formerly premature infants with bronchopulmonary dysplasia: clinical features and outcomes in the surfactant era. Pediatrics [Internet]. 2007 Dec [cited 2022 Jul 10];120(6):1260–9. Available from: https://pubmed.ncbi.nlm.nih.gov/18055675/18055675 10.1542/peds.2007-0971

[6] Altit G, Bhombal S, Hopper RK, Tacy TA, Feinstein J. Death or resolution: the “natural history” of pulmonary hypertension in bronchopulmonary dysplasia. J Perinatol [Internet]. 2019 Mar 1 [cited 2022 Jul 13];39(3):415–25. Available from: https://pubmed.ncbi.nlm.nih.gov/30617286/30617286 10.1038/s41372-018-0303-8

[7] Ong MS, Abman S, Austin ED, Feinstein JA, Hopper RK, Krishnan US, Racial and Ethnic Differences in Pediatric Pulmonary Hypertension: An Analysis of the Pediatric Pulmonary Hypertension Network Registry. J Pediatr [Internet]. 2019 Aug 1 [cited 2022 Jul 20];211:63–71.e6. Available from: https://pubmed.ncbi.nlm.nih.gov/31176455/31176455 10.1016/j.jpeds.2019.04.046 PMC6776463

[8] Arjaans S, Haarman MG, Roofthooft MTR, Fries MWF, Kooi EMW, Bos AF, Fate of pulmonary hypertension associated with bronchopulmonary dysplasia beyond 36 weeks postmenstrual age. Arch Dis Child Fetal Neonatal Ed. 2021;106(1):45–50. 32571832 10.1136/archdischild-2019-318531 PMC7788204

[9] Abman SH. Pulmonary Hypertension: The Hidden Danger for Newborns. Neonatology. 2021;118(2):211–7. 33951650 10.1159/000516107 PMC8177056

[10] Malloy KW, Austin ED. Pulmonary hypertension in the child with bronchopulmonary dysplasia. Pediatr Pulmonol [Internet]. 2021 Nov 1 [cited 2022 Jul 13];56(11):3546–56. Available from: https://pubmed.ncbi.nlm.nih.gov/34324276/34324276 10.1002/ppul.25602 PMC8530892

[11] Lagatta JM, Hysinger EB, Zaniletti I, Wymore EM, Vyas-Read S, Yallapragada S, The Impact of Pulmonary Hypertension in Preterm Infants with Severe Bronchopulmonary Dysplasia through 1 Year. Journal of Pediatrics. 2018;203:218–224.e3. 30172426 10.1016/j.jpeds.2018.07.035 PMC6460906

[12] Swier NL, Richards B, Cua CL, Lynch SK, Yin H, Nelin LD, Pulmonary Vein Stenosis in Neonates with Severe Bronchopulmonary Dysplasia. Am J Perinatol [Internet]. 2016 Jun 1 [cited 2022 Jul 19];33(7):671–7. Available from: https://pubmed.ncbi.nlm.nih.gov/26862723/10.1055/s-0035-1571201 26862723

[13] Abman SH, Hansmann G, Archer SL, Ivy DD, Adatia I, Chung WK, Pediatric pulmonary hypertension. Circulation. 2015;132(21):2037–99. 26534956 10.1161/CIR.0000000000000329

[14] Hansmann G, Koestenberger M, Alastalo TP, Apitz C, Austin ED, Bonnet D, 2019 updated consensus statement on the diagnosis and treatment of pediatric pulmonary hypertension: The European Pediatric Pulmonary Vascular Disease Network (EPPVDN), endorsed by AEPC, ESPR and ISHLT. Journal of Heart and Lung Transplantation. 2019;38(9):879–901. 10.1016/j.healun.2019.06.022 31495407

[15] Abman SH, Mullen MP, Sleeper LA, Austin ED, Rosenzweig EB, Kinsella JP, Characterisation of paediatric pulmonary hypertensive vascular disease from the PPHNet Registry. Eur Respir J [Internet]. 2021 Jan 1 [cited 2022 Jun 30];59(1). Available from: https://pubmed.ncbi.nlm.nih.gov/34140292/10.1183/13993003.03337-2020 PMC10335325 34140292

[16] Krishnan U, Feinstein JA, Adatia I, Austin ED, Mullen MP, Hopper RK, Evaluation and Management of Pulmonary Hypertension in Children with Bronchopulmonary Dysplasia. Journal of Pediatrics. 2017;188:24–34.e1. 28645441 10.1016/j.jpeds.2017.05.029

[17] Collaco JM, Abman SH, Austin ED, Avitabile CM, Bates A, Fineman JR, Kids Mod PAH trial: A multicenter trial comparing mono- versus duo therapy for initial treatment of pediatric pulmonary hypertension. Pulm Circ [Internet]. 2023 Oct 1 [cited 2024 Feb 17];13(4). Available from: /pmc/articles/PMC10617301/10.1002/pul2.12305 PMC10617301 37915400

[18] Abman SH, Collaco JM, Shepherd EG, Keszler M, Cuevas-Guaman M, Welty SE, Interdisciplinary Care of Children with Severe Bronchopulmonary Dysplasia. Journal of Pediatrics. 2017;181:12–28.e1. 27908648 10.1016/j.jpeds.2016.10.082 PMC5562402

[19] Awerbach JD, Mallory GB, Kim S, Cabrera AG. Hospital Readmissions in Children with Pulmonary Hypertension: A Multi-Institutional Analysis. Journal of Pediatrics. 2018;195:95–101.e4. 29336798 10.1016/j.jpeds.2017.11.027

[20] Bamat NA, Kirpalani H, Feudtner C, Jensen EA, Laughon MM, Zhang H, Medication use in infants with severe bronchopulmonary dysplasia admitted to United States children’s hospitals. Journal of Perinatology 2019 39:9 [Internet]. 2019 Jun 21 [cited 2024 Feb 15];39(9):1291–9. Available from: https://www.nature.com/articles/s41372-019-0415-9 10.1038/s41372-019-0415-9 PMC6713592 31227785

[21] Bamat NA, Nelin TD, Eichenwald EC, Kirpalani H, Laughon MM, Jackson WM, Loop Diuretics in Severe Bronchopulmonary Dysplasia: Cumulative Use and Associations with Mortality and Age at Discharge. J Pediatr [Internet]. 2021 Apr 1 [cited 2022 Sep 27];231:43–49.e3. Available from: https://pubmed.ncbi.nlm.nih.gov/33152371/33152371 10.1016/j.jpeds.2020.10.073 PMC8005411

[22] Backes CH, Reagan PB, Smith C V., Jadcherla SR, Slaughter JL. Sildenafil Treatment of Infants With Bronchopulmonary Dysplasia-Associated Pulmonary Hypertension. Hosp Pediatr. 2016;6(1):27–33. 26666265 10.1542/hpeds.2015-0076

[23] Greenberg JM, Poindexter BB, Shaw PA, Bellamy SL, Keller RL, Moore PE, Respiratory medication use in extremely premature (< 29 weeks) infants during initial NICU hospitalization: Results from the prematurity and respiratory outcomes program. Pediatr Pulmonol [Internet]. 2020 Feb 1 [cited 2022 Jun 7];55(2):360–8. Available from: https://onlinelibrary.wiley.com/doi/full/10.1002/ppul.24592 31794157 10.1002/ppul.24592

[24] Stenger MR, Slaughter JL, Kelleher K, Shepherd EG, Klebanoff MA, Reagan P, Hospital variation in nitric oxide use for premature infants. Pediatrics [Internet]. 2012 Apr [cited 2022 Jun 5];129(4):e945–51. Available from: http://www.ncbi.nlm.nih.gov/pubmed/22412028 22412028 10.1542/peds.2011-1809 PMC3313635

[25] Laas E, Lelong N, Thieulin AC, Houyel L, Bonnet D, Ancel PY, Preterm birth and congenital heart defects: a population-based study. Pediatrics [Internet]. 2012 Oct [cited 2023 Nov 27];130(4). Available from: https://pubmed.ncbi.nlm.nih.gov/22945415/10.1542/peds.2011-3279 22945415

[26] Jensen EA, Dysart K, Gantz MG, McDonald S, Bamat NA, Keszler M, The Diagnosis of Bronchopulmonary Dysplasia in Very Preterm Infants An Evidence-based Approach. Am J Respir Crit Care Med. 2019;200(6):751–9. 30995069 10.1164/rccm.201812-2348OC PMC6775872

[27] Fenton TR, Kim JH. A systematic review and meta-analysis to revise the Fenton growth chart for preterm infants. BMC Pediatr [Internet]. 2013 Apr 20 [cited 2022 Sep 13];13(1):1–13. Available from: https://bmcpediatr.biomedcentral.com/articles/10.1186/1471-2431-13-59 23601190 10.1186/1471-2431-13-59 PMC3637477

[28] Baum CF. Stata tip 63: Modeling proportions [Internet]. Vol. 8, The Stata Journal. 2008. Available from: http://www.stata-press.com/data/r10/census7

[29] Humpl T, Reyes JT, Erickson S, Armano R, Holtby H, Adatia I. Sildenafil therapy for neonatal and childhood pulmonary hypertensive vascular disease. Cardiol Young [Internet]. 2011 Apr [cited 2022 Jul 19];21(2):187–93. Available from: https://pubmed.ncbi.nlm.nih.gov/21138617/21138617 10.1017/S1047951110001745

[30] Mourani PM, Sontag MK, Ivy DD, Abman SH. Effects of long-term sildenafil treatment for pulmonary hypertension in infants with chronic lung disease. J Pediatr [Internet]. 2009 [cited 2022 Jul 19];154(3). Available from: https://pubmed.ncbi.nlm.nih.gov/18950791/10.1016/j.jpeds.2008.09.021 PMC2783835 18950791

[31] Wardle AJ, Wardle R, Luyt K, Tulloh R. The utility of sildenafil in pulmonary hypertension: a focus on bronchopulmonary dysplasia. Arch Dis Child [Internet]. 2013 Aug [cited 2022 Jul 19];98(8):613–7. Available from: https://pubmed.ncbi.nlm.nih.gov/23625986/23625986 10.1136/archdischild-2012-303333

[32] Baquero H, Soliz A, Neira F, Venegas ME, Sola A. Oral sildenafil in infants with persistent pulmonary hypertension of the newborn: a pilot randomized blinded study. Pediatrics [Internet]. 2006 [cited 2022 Jul 19];117(4):1077–83. Available from: https://pubmed.ncbi.nlm.nih.gov/16585301/16585301 10.1542/peds.2005-0523

[33] Schneider S, Bailey M, Spears T, Esther CR, Laughon MM, Hornik CP, Safety of sildenafil in premature infants with severe bronchopulmonary dysplasia (SILDI-SAFE): a multicenter, randomized, placebo-controlled, sequential dose-escalating, double-masked, safety study. BMC Pediatr [Internet]. 2020 Dec 1 [cited 2022 Jul 17];20(1):1–9. Available from: https://bmcpediatr.biomedcentral.com/articles/10.1186/s12887-020-02453-7 33317479 10.1186/s12887-020-02453-7 PMC7735412

[34] Lang JE, Hornik CD, Martz K, Jacangelo J, Anand R, Greenberg R, Safety of sildenafil in premature infants at risk of bronchopulmonary dysplasia: Rationale and methods of a phase II randomized trial. Contemp Clin Trials Commun [Internet]. 2022 Dec 1 [cited 2024 Feb 17];30. Available from: https://pubmed.ncbi.nlm.nih.gov/36345347/10.1016/j.conctc.2022.101025 PMC9636444 36345347

[35] Hasan SU, Potenziano J, Konduri GG, Perez JA, Van Meurs KP, Walker MW, Effect of Inhaled Nitric Oxide on Survival Without Bronchopulmonary Dysplasia in Preterm Infants: A Randomized Clinical Trial. JAMA Pediatr [Internet]. 2017 Nov 1 [cited 2023 Nov 26];171(11):1081–9. Available from: https://pubmed.ncbi.nlm.nih.gov/28973344/28973344 10.1001/jamapediatrics.2017.2618 PMC5710365

[36] Barrington KJ, Finer N, Pennaforte T. Inhaled nitric oxide for respiratory failure in preterm infants. Vol. 2017, Cochrane Database of Systematic Reviews. John Wiley and Sons Ltd; 2017. 10.1002/14651858.CD000509.pub5 PMC6464861 28045472

[37] Thompson EJ, Perez K, Hornik CP, Smith PB, Clark RH, Laughon M. Sildenafil Exposure in the Neonatal Intensive Care Unit. Am J Perinatol [Internet]. 2019 [cited 2024 Apr 18];36(3):262. Available from: /pmc/articles/PMC6996478/30081404 10.1055/s-0038-1667378 PMC6996478

[38] Barrington KJ, Finer N, Pennaforte T, Altit G. Nitric oxide for respiratory failure in infants born at or near term. Cochrane Database of Systematic Reviews [Internet]. 2017 Jan 5 [cited 2024 Apr 18];2017(1). Available from: https://www.cochranelibrary.com/cdsr/doi/10.1002/14651858.CD000399.pub3/full 10.1002/14651858.CD000399.pub3 PMC6464941 28056166

[39] van der Graaf M, Rojer LA, Helbing WA, Reiss IKM, Etnel JRG, Bartelds B. Sildenafil for bronchopulmonary dysplasia and pulmonary hypertension: a meta-analysis. Pulm Circ [Internet]. 2019 Jul 1 [cited 2024 Feb 17];9(3). Available from: /pmc/articles/PMC6681505/10.1177/2045894019837875 PMC6681505 30803328

[40] Jackson W, Gonzalez D, Smith PB, Ambalavanan N, Atz AM, Sokol GM, Safety of sildenafil in extremely premature infants: a phase I trial. Journal of Perinatology 2021 42:1 [Internet]. 2021 Nov 5 [cited 2024 Jan 28];42(1):31–6. Available from: https://www.nature.com/articles/s41372-021-01261-w 10.1038/s41372-021-01261-w PMC8569839 34741102

[41] Mourani PM, Sontag MK, Younoszai A, Miller JI, Kinsella JP, Baker CD, Early pulmonary vascular disease in preterm infants at risk for bronchopulmonary dysplasia. Am J Respir Crit Care Med [Internet]. 2015 Jan 1 [cited 2022 Jun 26];191(1):87–95. Available from: https://pubmed.ncbi.nlm.nih.gov/25389562/25389562 10.1164/rccm.201409-1594OC PMC4299632

[42] Arjaans S, Fries MWF, Schoots MH, Schilte CFM, Roofthooft MTR, Vrijlandt EJLE, Clinical Significance of Early Pulmonary Hypertension in Preterm Infants. J Pediatr. 2022;251:74–81.e3. 35934129 10.1016/j.jpeds.2022.07.039

[43] Lewis TR, Kielt MJ, Walker VP, Levin JC, Guaman MC, Panitch HB, Association of Racial Disparities With In-Hospital Outcomes in Severe Bronchopulmonary Dysplasia. JAMA Pediatr [Internet]. 2022;176(9):852–9. Available from: 10.1001/jamapediatrics.2022.2663 35913704 PMC9344383

[44] Backes CH, Hill KD, Shelton EL, Slaughter JL, Lewis TR, Weisz DE, Patent Ductus Arteriosus: A Contemporary Perspective for the Pediatric and Adult Cardiac Care Provider. J Am Heart Assoc [Internet]. 2022;11(17):e025784. Available from: 10.1161/JAHA.122.025784 36056734 PMC9496432

